# Selective Inhibitors of Kv11.1 Regulate IL-6 Expression by Macrophages in Response to TLR/IL-1R Ligands

**DOI:** 10.1100/tsw.2010.155

**Published:** 2010-08-17

**Authors:** Cheryl Hunter, Tejas B. Kadakia, Dianne Cooper, Mauro Perretti, Richard C. Schwartz, Simon B. Brown

**Affiliations:** ^1^ MRC Centre for Inflammation Research, Queen's Medical Research Institute, College of Medicine and Veterinary Medicine, University of Edinburgh, UK; ^2^ Department of Microbiology and Molecular Genetics, Michigan State University, East Lansing, MI, USA; ^3^ William Harvey Research Institute, Barts and The London School of Medicine, Queen Mary University of London, UK

**Keywords:** PECAM-1, endotoxin, cytokines, macrophages, inflammation

## Abstract

The mechanism by which the platelet-endothelial cell adhesion molecule PECAM-1 regulates leukodiapedesis, vascular endothelial integrity, and proinflammatory cytokine expression *in vivo* is not known. We recently identified PECAM-1 as a negative regulator of Kv11.1, a specific voltage-gated potassium channel that functioned in human macrophages to reset a resting membrane potential following depolarization. We demonstrate here that dofetilide (DOF), a selective inhibitor of the Kv11.1 current, had a profound inhibitory effect on neutrophil recruitment in mice following TLR/IL-1R–elicited peritonitis or intrascrotal injection of IL-1β, but had no effect on responses seen with TNFα. Furthermore, inhibitors of Kv11.1 (DOF, E4031, and astemizole), but not Kv1.3 (margatoxin), suppressed the expression of IL-6 and MCP-1 cytokines by murine resident peritoneal macrophages, while again having no effect on TNFα. In contrast, IL-6 expression by peritoneal mesothelial cells was unaffected. Using murine P388 cells, which lack endogenous C/EBPβexpression and are unresponsive to LPS for the expression of both IL-6 and MCP-1, we observed that DOF inhibited LPS-induced expression of IL-6 mRNA following ectopic expression of wild-type C/EBPβ, but not a serine-64 point mutant. Finally, DOF inhibited the constitutive activation of cdk2 in murine peritoneal macrophages; cdk2 is known to phosphorylate C/EBPβ at serine-64. Taken together, our results implicate a potential role for Kv11.1 in regulating cdk2 and C/EBPβ activity, where robust transactivation of both IL-6 and MCP-1 transcription is known to be dependent on serine-64 of C/EBPβ. Our data might also explain the altered phenotypes displayed by PECAM-1 knockout mice in several disease models.

### INTRODUCTION

Leukodiapedesis is a complex series of biological processes whereby leukocytes are captured from the circulation and transmigrate across both the endothelium and perivascular basement membrane to a site of tissue injury[1,2,3]. PECAM-1, a platelet-endothelial cell adhesion molecule, also referred to as CD31 and expressed by leukocytes, platelets, and endothelial cells, is a key regulator of leukodiapedesis[1,2,3]. Although the exact mechanism by which PECAM-1 regulates leukodiapedesis is not known, evidence exists to suggest that homophilic ligation of PECAM-1 can regulate β1, β2, and β3 integrin function in promoting cell-cell and/or cell-matrix interactions[3].

We recently reported the novel and unexpected finding that Kv11.1, a specific voltage-gated potassium channel also known as ERG (ether-a-go-go related gene product), was a downstream effector of PECAM-1–dependent signaling in regulating both macrophage recognition of apoptotic leukocytes[4,5,6] and leukocyte recruitment from the circulation[7]. In particular, we found that Kv11.1 acted downstream of macrophage PECAM-1 to regulate β1 integrins in promoting the binding and engulfment of apoptotic cells *in vitro*[6,8]. It is of further interest that Kv11.1 is known to form a macromolecular complex with β1 integrins in various cancer cell lines[8,9].

Kv11.1 is better known for its presence in excitable cells, where it is involved in the repolarization of the cardiac action potential and in spike-frequency adaptation of neuroendocrine cells[10,11]. Kv11.1, however, has also been described in nonexcitable cells, including human monocyte-derived macrophages and rat microglia, tissue-resident macrophages of the brain[6,12,13,14]. Within the macrophage, and analogous with its role in cardiomyocytes, Kv11.1 functioned to re-establish a resting membrane potential following membrane depolarization[6,13]. Depolarization can occur when macrophages bind apoptotic cells[6,15], and repolarization wasdelayed when macrophage PECAM-1 was ligated by PECAM-1 presented on apoptotic cells[6,8]. Importantly, depolarization favored integrin-mediated adhesive events.

PECAM-1 is a type-I transmembrane protein and a member of the immunoglobulin (Ig) superfamily comprising an extracellular domain of six C2-type Ig domains (D1–6) and a cytoplasmic tail of 118 amino acids[16]. Although PECAM-1 contains no intrinsic kinase or phosphatase activity, its intracellular C-terminal tail is phosphorylated on serine, threonine, and tyrosine residues in response to receptor engagement and a variety of other stimuli[3]. Of these residues, tyrosines Y663 and Y686 comprise an immunomodulatory tyrosine-based inhibitor motif (ITIM) which, when phosphorylated, recruits the nonreceptor tyrosine phosphatases SHP-1 or SHP-2[17,18]. The binding of SHP-1 is often invoked to explain how PECAM-1 functions as a negative regulator of T-cell, B-cell, mast cell, and platelet activity[18]. In contrast, we demonstrated that the ITIM of PECAM-1 ectopically expressed in K562 cells was dispensable for regulating an endogenous Kv11.1 current, and that an intact full-length cytoplasmic tail was essential for the binding and engulfment of apoptotic leukocytes by COS-7 cells[5,6].

PECAM-1 is reported also to inhibit the production of proinflammatory cytokines in several rodent models of disease[19,20,21,22]. Of particular relevance, PECAM-1 knockout (–/–) mice are more susceptible to endotoxic shock and nonalcoholic steatohepatitis, in both cases exhibiting elevated plasma concentrations of proinflammatory cytokines[19,20,23]. In contrast, macrophage expression of PECAM-1 appears to be obligatory for both TNFα cytokine production and leukocyte recruitment in response to immune complex deposition within the lung[22]. Precisely how PECAM-1 can regulate cytokine production, either positively or negatively, is still unclear.

Here we demonstrate that chemical inhibitors with well-characterized selectivity for Kv11.1 activity have a significant inhibitory effect on both IL-6 cytokine expression and leukocyte recruitment in sterile models of peritonitis. We further provide evidence that exposure of resident peritoneal macrophages *in vitro* with selective inhibitors of the Kv11.1 current can suppress IL-6 expression in a cyclin-dependent kinase-2– (cdk2) (a Ser/Thr protein kinase) and CCAAT/enhancer binding protein beta– (C/EBPβ) dependent manner. We suggest that by modifying the dynamics of macrophage membrane repolarization, PECAM-1 cannot only influence apoptotic cell binding, but also LPS- and IL-1β–induced IL-6 expression, which could explain the altered phenotypes displayed by PECAM-1 –/– mice in several disease models.

## MATERIALS AND METHODS

### Reagents

Endothelial basal medium-2 (EBM2) and growth supplements were obtained from Clonetics (BioWhittaker, Wokingham, U.K.). Penicillin G, streptomycin sulfate, DMEM, and IMDM were obtained from Life Technologies (Paisley, U.K.). Antibodies to cdk2 (clone 78B2) and phospho-CDK2(Thr160)(#2561S) were from Cell Signaling Technology (Danvers, MA). Antibody to phospho-C/EBPβ (Ser64) was a gift from Dr. Peter Johnson (National Cancer Institute, Frederick, MD). Antibodies to Kv11.1 were from Abcam (ab85398) (www.abcam.com), Alamone (APC-062)(www.alamone.com), and Chemicon (AB9796)(www.chemicon.com).

### Mice

IL-6 (male and female) and PECAM-1 (female) –/– mice on a C57BL/6 background, as well as wild-type C57BL/6 mice (female, B&K Universal, Hull, U.K.), were kept in specific pathogen-free conditions and were maintained on a standard chow pellet diet with tap water *ad libitum*. In all cases, animals were housed at a density of five animals per cage in a room with controlled lighting (lights on from 8:00 a.m. to 8:00 p.m.), in which the temperature was maintained at 21–23°C. All animal experiments were performed on 8- to 12-week-old mice according to institutional guidelines and U.K. Home Office regulations.

### Peritonitis

Peritonitis was induced by i.p. injection of 300 μL of either Brewer's thioglycollate (BTG) (10% w/v), IL-1β (33 ng/mL), TNFα (125 ng/mL), or LPS (1 μg/mL) in mice that had been i.p. injected 10 min earlier with either 200 μL of PBS or PBS containing dofetilide (DOF) at the desired concentration. In some experiments, a soluble gp130 (250 ng/mouse; R&D Systems, Minneapolis, MN) was coadministered with the PBS ± DOF. Mice underwent peritoneal lavage at either 1 or 4 h with 5.0 mL of ice-cold sterile PBS using a 19-gauge needle (average volume recovery 4.3 mL).

### Intravital Microscopy of the Mouse Cremaster Muscle

Mice were injected with DOF (10 mg/kg) or vehicle control (i.p.) 10 min prior to intrascrotal injection of IL-1β (30 ng) or TNFα (300 ng). The cremaster was prepared for intravital microscopy as described previously[24] such that observations were recorded 4 h postinjection. Briefly, mice were anesthetized with a mixture of xylazine (7.5 mg/kg) and ketamine (150 mg/kg), the cremaster was then dissected free of skin and fascia, opened longitudinally, and pinned against the viewing platform of a plexiglass stage. The preparation was mounted on a Zeiss Axioskop "FS" microscope (original magnification; 40×; Carl Zeiss, Welwyn Garden City, U.K.) and transilluminated with a 12-V, 100-W halogen light source. To avoid drying out, the cremaster muscle was superfused with bicarbonate-buffered saline (pH 7.4, 37°C, gassed with 5% $CO_2$/95% N2) at a rate of 2 mL/min.

Following a 30-min stabilization period, video recordings were made with a Hitachi CCD color camera (KPC571; Tokyo, Japan) and an S-VHS video recorder (SVO-9500MDP) for subsequent off-line analysis. Leukocyte rolling flux, firm adhesion, and transmigration in postcapillary venules with a wall shear rate ~500/sec and diameter 20–40 μm were quantified as previously described[24]. Briefly, rolling flux was quantified as the number of rolling leukocytes to pass a defined point on the venular wall per minute. In each animal, responses from several vessel segments (3–5) and multiple vessels (3–5) were quantified.

### Tissue Culture of Peritoneal Cells

Peritoneal lavages from naïve mice were pooled, collected by centrifugation at 300 × *g*, and the cell pellet resuspended in DMEM/F12 before plating out in 48-well plates at $2 \times 10^5$ cells per well. Following incubation for 1 h at 37°C and 5% $CO_2$, nonadherent cells were aspirated and the medium replaced with DMEM/F12 supplemented with 500 U/mL penicillin, 500 U/mL streptomycin, and 10% FCS for 24 h. The adherent cells, comprised of tissue-resident peritoneal macrophages (RPMΦ), were then washed with HBSS before leaving in DMEM/F12 without any growth supplements in which DOF (10 μM) may have been added. After 30 min in serum-free DMEM/F12, cells were exposed to either BTG (1% final), LPS (10 ng/mL), IL-1β (10 ng/mL), or TNFα (40 ng/mL) for either 1, 4, or 21 h, after which supernatants were collected and stored frozen at –70°C until analyzed.

Mesothelial cells were isolated from resected mesentery following enzymatic disaggregation with trypsin/EDTA solution. After 10 min of incubation, the mesentery was removed and a culture medium consisting of Medium199:MCDB105 (1:1 vol/vol) supplemented with FCS (15% vol/vol), streptomycin (50 μg/mL), penicillin (50 IU/mL), and L-glutamine (2 mmol/L) was added. Cells were washed with HBSS and seeded into collagen type-I–coated 24-well plates (CellCoat, GreinerBio-One, U.K.) before being incubated at 37°C in a humidified incubator under an atmosphere of 95% air, 5% $CO_2$ for 24 h. Adherent cells were gently washed of conditioned media and exposed to 1% BTG in DMEM/F12 supplemented with 500 U/mL penicillin, 500 U/mL streptomycin, and 10% FCS in either the presence or absence of DOF for a further 21 h.

### Fluorescence Imaging and Flow Cytometry

Peritoneal cell preparations were assessed for purity by epifluorescent microscopy and flow cytometry using either an Axioskop II MOT microscope (Zeiss) fitted with an ORCA camera or a FACSCalibur flow cytometer (BD Biosciences, www.bdbiosciences.com). Cells either in suspension unfixed (flow cytometry) or adherent to glass coverslips (epifluorescent imaging) and fixed with 2% paraformaldehyde were blocked in PBS containing 5% rat and 5% hamster serum before addition of either anti-CD11b (clone M1/70; BD Biosciences), anti-CD19 (1D3; BD Biosciences), anti-Gr1 (RB6-8C5; eBioscience, www.ebioscience.com), anti-CD117 (2B8; eBioscience), or anti-F4/80 (CI:A3-1; Caltag, www.invitrogen.com). All antibodies were primary conjugated with either FITC or R-PE, and were applied at 5 μg/mL or less for 30 min before washing and analysis. Appropriate isotype antibody controls were used in all instances.

### Real-Time PCR

Template cDNA for RT-PCR was generated using the Transcriptor First Strand cDNA Synthesis kit (Roche, www.roche.com). Standard PCR reactions were carried out using Promega’s (www.promega.com) PCR master mix (M7502), primers at a final concentration of 20 nM each and 35 cycles. Quantitative PCR was carried out using Applied Biosystems TaqMan (www.appliedbiosystems.com) reaction master mix, 18S internal control, and TaqMan gene expression sets following the manufacturer’s recommended procedures on an Applied Biosystems ABI 7900 machine. Kv11.1 was assessed with the TaqMan primer set Mm00465370_m1.

### RNA Protection Assays

P388-Cβ cells[25] and P388-CβS64A cells[26] were cultured in RPMI 1640 medium supplemented to 5% FCS and 50 μM 2-ME. IL-6 induction was conducted with LPS derived from *Escherichia coli*

serotype 055:B5 (Sigma, St. Louis, MO) added to 10 µg/mL. Some cells were pretreated with 10 µM DOF for 10 min prior to LPS induction. Total RNA was isolated over a time course of 0, 8, and 24 h using TRIzol reagent (Invitrogen, Carlsbad, CA) according to the manufacturer's directions. Synthesis of cDNA was performed by reverse transcription with 2 µg of total RNA using the Superscript II kit with oligo dT(12-18) primers as described by the manufacturer (Invitrogen). cDNA (1 µL) was amplified by PCR in a final volume of 25 µL using the iQ SYBR Green Supermix (Bio-Rad, Hercules, CA) with 10 pmol of each primer. IL-6 was amplified using 5 -ATC CAG TTG CCT TCT TGG GAC TGA-3 and 5 -TAA GCC TCC GAC TTG TGA AGT GGT-3. HPRT was also used as a nonmodulated control gene and was amplified using 5 -AAG CCT AAG ATG AGC GCA AG-3 and 5 -TTA CTA GGC AGA TGG CCA CA-3. Real-time PCR was carried out for 40 cycles using the iCycler (Bio-Rad) and data were evaluated using the iCycler software. Each cycle consisted of 95°C for 15 sec, 60°C for 30 sec, and 72°C for 30 sec. RNA-free samples, a negative control, were not amplified. Reactions lacking reverse transcriptase also were not amplified. Melting curve and gel analyses were used to verify single products of the appropriate base pair size as described[27].

### Cytokine Bead Array and ELISA

Cytokines were analyzed either with a murine inflammation Cytokine Bead Array kit (Becton-Dickinson, www.bd.com) or ELISA using DuoSet kits (R&D Systems) according to the manufacturers' instructions.

### Cell Lysis, SDS-PAGE, and Western Blotting

RPMΦ were lysed in 10 mM Hepes buffer, pH 7.4, containing 1% Triton-X100, 1 mM EDTA, 1 mM benzamidine, 10 µM pepstatin A, 1 mM σ-phenanthroline, 10 µM leupeptin, 1 mM phenylmethylsulfonylfluoride, 1 mM sodium vanadate, and 1 mM sodium fluoride. Supernatants were collected following centrifugation at 12,000 × *g* before diluting with a 4x Laemmli buffer. Each sample was prepared on the basis of equal numbers of extracted cells rather than on protein content. SDS minislab gels (10 × 8 $cm^2$) were prepared and run according to the method of Laemmli, except the anode buffer contained no glycine. Transfer of proteins to nitrocellulose was performed at 0.5 $mA/cm^2$ for 2 h at 4°C and visualized by Ponceau Red. Membranes were blocked in 4% (w/v) milk powder/PBS (Marvel, 99% fat-free) containing 0.1% Tween-20 before adding primary antibody at 1:2000 overnight where all steps were performed at 4°C. Blots were then washed with PBS before probing with a peroxidise-conjugated secondary pAb of appropriate specificity with detection by digital camera using a versadoc system and SuperSignal West Femto detection reagents (Thermo Scientific, www.thermo.com). Quantitation of protein bands by Western blot was performed with Quantity One software (BioRad, version 4.5.0), where total C/EBPβ levels were corrected for loading relative to a β-actin control (clone AC-15).

For the analysis of C/EBPβ serine-64 phosphorylation, P388 cells were seeded at 1 × $10^6$ cells per 6-cm dish and transfected with 5 µg of C/EBPβ expression plasmid using DMRIE-C reagent (Invitrogen) according to the manufacturer's recommendations. The cells were treated with LPS (10 µg/mL) 48 h post-transfection and, in some instances, pretreated with 10 µM DOF for 10 min prior to LPS induction. Cells were lysed in RIPA buffer at various time points. Extracts were processed by SDS-12% PAGE, transferred to a Protran membrane (Whatman, Florham Park, NJ), and C/EBPβ phosphorylation at serine-64 was analyzed as described[26]. Protein bands were quantitated with NIH Image J software (Rasband WS. ImageJ. National Institutes of Health, Bethesda, MD. Available from: http://rsb.info.nih.gov/ij/).

### Statistical Analysis

All values are expressed as mean ± 95% CI (confidence interval) unless stated otherwise with the number (n) of animals or experiments defined elsewhere. Statistical analysis was assessed either by Student's t-test or by one-way ANOVA followed by Bonferroni, Tukey, or Scheffé posthoc tests as appropriate.

## RESULTS

### Dofetilide is Anti-Inflammatory in a Cremasteric Model of Inflammation

We have shown previously that human monocyte-derived macrophages express Kv11.1, that PECAM-1 can regulate Kv11.1 activity in the erythroleukemic cell line K562, and that class III antiarrhythmic methanesulfonanilides that are selective for inhibiting the Kv11.1 current are anti-inflammatory in a zebrafish model of inflammation[6,7,12]. To test whether a drug of this class, DOF, could act similarly in a murine model of acute inflammation in the context of a PECAM-1–dependent response, we employed intravital microscopy and intrascrotal injection of either IL-1β or TNFα, and monitored leukocyte behavior[24]. In these models, DOF inhibited the adhesion and transmigration, but not the rolling, of leukocytes across postcapillary venules in response to IL-1β, whereas the response to TNFα was unaffected (Fig. 1). This apparent discordance in response between IL-1β and TNFα is identical to that seen for wild-type vs. PECAM-1 –/– mice, where the absence of PECAM-1 resulted in an approximately 55% reduction in the number of leukocytes that transmigrated in response to IL-1β[28].

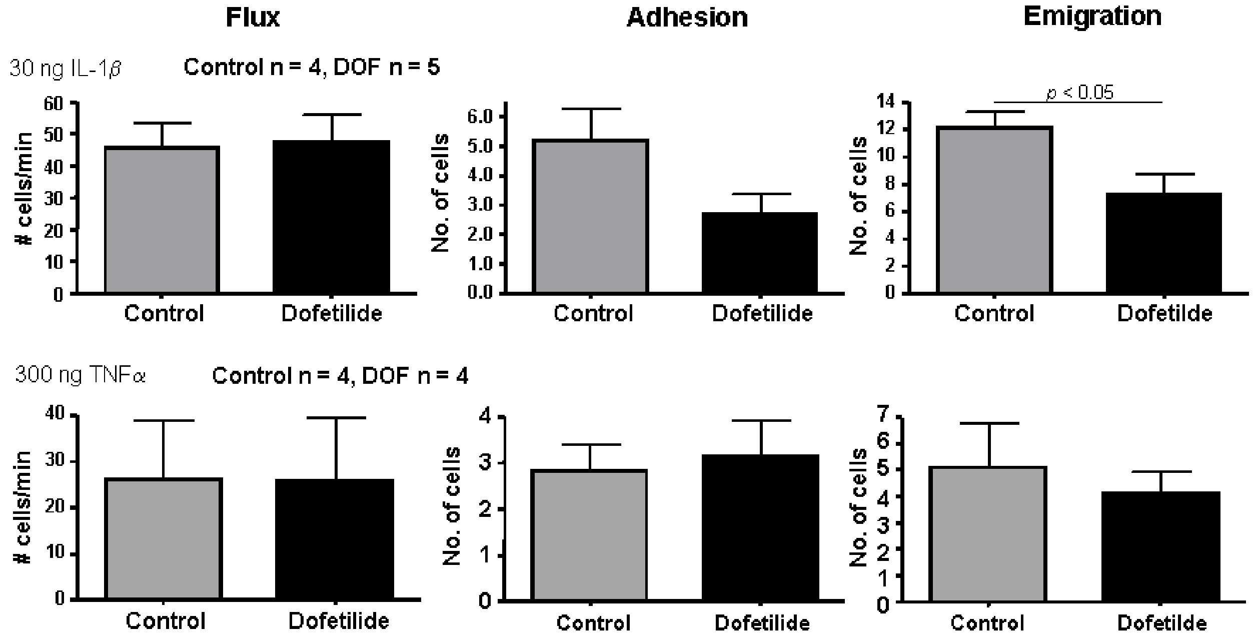

**FIGURE 1**. Effect of DOF on leukocyte rolling, adhesion, and emigration through IL-1β–stimulated mouse cremasteric venules. Animals were injected intrascrotally with either IL-1β (30 ng; top row) or TNFα (300 ng; bottom row) 4 h prior to externalization of the cremaster muscle for intravital microscopy in which mice had further been injected i.p. with either saline or DOF (10 mg/kg) 10 min prior to video recording. Data represent the mean ± SEM, where the number (n) of mice are defined within each panel.

### Dofetilide is Anti-Inflammatory in Several Models of Sterile Peritonitis

To expand the intravital microscopy observations to a more aggressive model of acute inflammation, the anti-inflammatory effects of DOF were tested in several murine models of sterile peritonitis[29]. Although the peritonitis model was not amenable for monitoring leukocyte rolling and adhesion to the luminal surface of the activated endothelium, as in the cremasteric model, it did allow assessment of leukocyte recruitment into the peritoneal cavity. Thus, we lavaged the peritonea of mice at 1 and 4 h after i.p. administration with known phlogistic agents, such as BTG, LPS, IL-1β, and TNFα. At these early time points and in agreement with the literature[30], the predominant infiltrating leukocyte was the $Gr1^+$ PMN (polymorphonuclear cells), while at the same time there was emigration of $F4/80^+$ tissue-resident macrophages (Table 1). Using wild-type C57BL/6 mice, we observed a dose-dependent DOF inhibition of tissue-resident macrophage emigration and PMN recruitment to the peritoneum in response to several proinflammatory stimuli, including IL-1β, but not TNFα (Table 1). In contrast, DOF had no effect in PECAM-1 –/– mice when BTG was used as the agonist. Similar results were obtained when DOF was administered in the drinking water of mice for 48 h prior to i.p. injection of BTG; e.g., $5.54 \pm 1.23 \times 10^6$ PMN were recovered from the peritonea of mice after 4 h in which mice drank daily on average 4.3 mL (untreated) vs. 4.5 mL (DOF, 1 g/L) and weighed on average 22.3 g.

**TABLE 1**
**Effect of DOF on Sterile Peritonitis***

| INSULT | DOF (mg/kg) | LAVAGE Time (h) | Cell Recovered ($x10^{-6}$) | n | F4/80 hi Mac | F4/80 lo Mono | $Gr1^+$ PMN |
|---|---|---|---|---|---|---|---|
| | | | wild-type | | | | |
| PBS | 0 | 0 | 1.25 ± 0.37 | 9 | 1.04 ± 0.28 | 0.02 ± 0.02 | <0.01 |
| BTG | 0 | 1 | 3.44 ± 1.76 | 5 | 0.71 ± 0.37 | 0.25 ± 0.17 | 2.38 ± 0.86 |
| BTG | 0 | 4 | 16.47 ± 4.24 | 12 | 0.09 ± 0.07 | 0.85 ± 0.43 | 13.30 ± 3.91 |
| BTG | 1 | 4 | 10.24 ± 3.73 | 5 | 0.19 ± 0.11 | 0.67 ± 0.23 | 7.18 ± 3.34 |
| BTG | 5 | 4 | 2.24 ± 1.93 | 5 | 0.76 ± 0.11 | 0.31 ± 0.23 | 1.27 ± 1.19 |
| BTG | 10 | 4 | 1.93 ± 0.81 | 8 | 0.89 ± 0.21 | 0.09 ± 0.10 | 0.87 ± 0.74 |
| LPS | 0 | 4 | 10.53 ± 3.66 | 5 | 0.18 ± 0.15 | 0.77 ± 0.27 | 7.64 ± 2.93 |
| LPS | 10 | 4 | 1.88 ± 0.72 | 6 | 0.68 ± 0.20 | 0.09 ± 0.09 | 0.92 ± 0.81 |
| IL-1β | 0 | 4 | 6.68 ± 2.87 | 5 | 0.14 ± 0.14 | 0.76 ± 0.33 | 4.67 ± 2.64 |
| IL-1β | 10 | 4 | 1.73 ± 0.92 | 5 | 1.09 ± 0.31 | 0.02 ± 0.03 | 0.55 ± 0.82 |
| TNFα | 0 | 4 | 5.33 ± 0.66 | 5 | 0.28 ± 0.44 | 0.93 ± 0.46 | 2.37 ± 0.79 |
| TNFα | 10 | 4 | 4.98 ± 1.02 | 5 | 0.48 ± 0.27 | 0.70 ± 0.44 | 3.12 ± 0.65 |
| | | | CD31–/– | | | | |
| PBS | 0 | 0 | 0.97 ± 0.14 | 6 | 0.84 ± 0.24 | 0.04 ± 0.02 | 0.06 ± 0.03 |
| BTG | 0 | 4 | 13.53 ± 2.81 | 6 | 0.14 ± 0.09 | 0.74 ± 0.23 | 11.14 ± 2.61 |
| BTG | 10 | 4 | 11.82 ± 3.73 | 9 | 0.18 ± 0.27 | 1.01 ± 0.31 | 10.15 ± 2.35 |

* Peritonitis and peritoneal lavages were performed as described in the text. Lavaged cells were enumerated by hemocytometer and flow cytometry. For flow cytometry, samples were spiked with a fixed quantity of 20-μm latex beads. Flow cytometric analysis also allowed quantitation of macrophages (Mac), which stained strongly positive for F4/80, monocytes (Mono), which stained weakly for F4/80, and PMN, which stained positive for Gr1.

### Inhibiting Kv11.1 Suppresses IL-6 Expression by Tissue-Resident Macrophages

Strikingly, wild-type mice receiving 10 mg/kg DOF i.p. failed to elicit an inflammatory response to BTG, with lavaged samples being identical to untreated controls with respect to resident macrophage numbers and the relative absence of infiltrated PMNs (Table 1). Clarified peritoneal lavage fluid was therefore analyzed by either cytokine bead array assay or ELISA to measure cytokine levels, including those of TNFα, IFNγ, IL-6, IL-10, IL-12p40, MCP-1, KC, and MIP-2. Although a significant reduction in the inducible expression of IL-6, TNFα, and IL-10 were observed with DOF at 10 mg/kg after 1-h exposure to BTG, only IL-6 ($p < 0.02$) and MCP-1 ($p < 0.1$) were found to be significantly inhibited after 4 h (Fig. 2). IFNγ and IL-12p40 were below the threshold of reliable detection, while KC (585 ± 133 pg/mL) and MIP-2 (743 ± 204 pg/mL) were unaffected. These results suggest that DOF can selectively regulate the inducible secretion of IL-6 and MCP-1 within the peritoneal cavity.

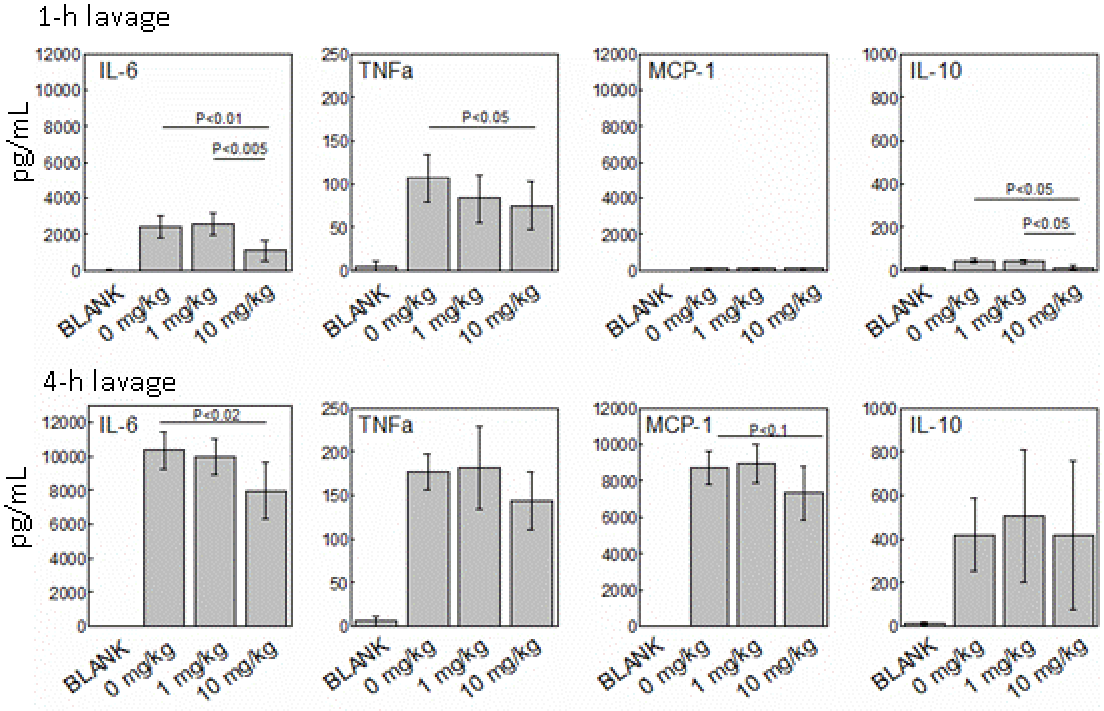

**FIGURE 2**. Levels of inflammatory cytokines in the murine-inflamed peritoneum. Lavages from the peritoneum of mice were analyzed by FACSArray bioanalyzer using a murine inflammation CBA kit for the detection of IL-6, TNFα, MCP-1, and IL-10. Mice were injected i.p with 200 µL of saline adjusted for whole body weight to deliver 0, 1, or 10 mg/kg of DOF followed 10 min later with 300 µL of 10% BTG. Lavages were performed with 5 mL PBS at 1 h (top row) or 4 h (bottom row). BLANK refers to naïve mice that received no injections. Data represent the mean ± 95% CI of five mice. Comparisons are only made for mice injected i.p. with BTG where cytokine levels were all significantly different from naïve mice, with the exception of IL-10 at 1 h for mice given DOF at 10 mg/kg.

Knowing that mesothelial cells and tissue-resident macrophages both respond to proinflammatory mediators by making IL-6, we decided to isolate murine mesothelial cells and RPMΦ from naïve peritonea. RPMΦ are isolated as a homogeneous preparation following the adhesion of peritoneal lavaged cells to glass or plastic surfaces where we typically only observe CD11b- and F4/80-positive cells as confirmed by immunofluorescence staining. Importantly, and after assessing over 24 × 13 mm coverslips from no less than four separate peritoneal lavages, each coverslip seeded with $1 \times 10^6$ cells, we failed to identify a single CD19-positive B cell, Gr1-positive PMN, or c-kit (CD117)–positive mast cell, despite their relative abundance in the lavage as confirmed by flow cytometry. Similarly, we found no evidence of CD11b-, F4/80-, Gr1-, CD19-, or CD117-positive cells within our mesothelial cell preparations. By plating out equivalent numbers of RPMΦ or mesothelial cells, and then stimulating with BTG, we observed a profound inhibitory effect of DOF (10 μM) on the ability of macrophages to produce IL-6, but not TNFα, over a 21-h time period (Fig. 3). In contrast, DOF (10 μM) had no effect on the ability of mesothelial cells to produce IL-6 in response to BTG. These results suggest that DOF affected the ability of RPMΦ to secrete inducibly, and presumably express, IL-6 *in vivo*. These results also suggest that any mesothelial cell contamination of the macrophage preparation was not a confounding variable.

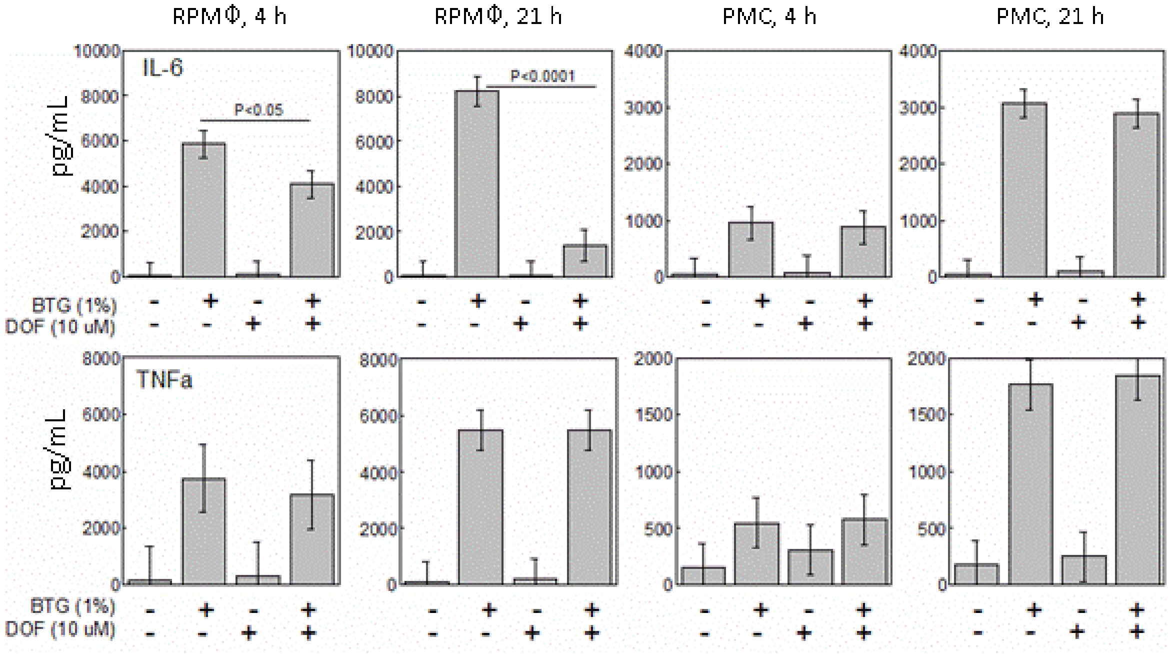

**FIGURE 3.** Effect of DOF on IL-6 and TNFα secretion by murine RPMΦ and peritoneal mesothelial cells (PMC) cultured *in vitro*. Supernatants were collected from RPMΦ and PMC cells exposed to either 1% BTG and/or DOF (10 μM) for either 4 or 21 h. Levels of IL-6 (top row) and TNFα (bottom row) were determined by FACSArray bioanalyzer using a murine inflammation CBA kit. Data represent the mean ± 95% CI of five independent experiments, each performed in triplicate.

To corroborate the inhibitory effect of DOF on IL-6 secretion by peritoneal macrophages, we repeated the above *in vitro* experiments with BTG, LPS, IL-1β, and TNFα as proinflammatory stimuli and measured IL-6 expression by ELISA. In keeping with the results obtained by cytokine bead array, DOF inhibited IL-6 expression by RPMΦ in response to 1% BTG and LPS (Fig. 4a). This inhibitory effect of DOF on IL-6 induction was also observed for IL-1β, but not TNFα (Fig. 4b). With IL-1β stimulation, we also found that alternative inhibitors of Kv11.1, but not an inhibitor of Kv1.3, could also suppress IL-6 expression (Fig. 4b). E4031 is selective for Kv11.1, while astemizole can also inhibit both Kv10.1, a closely related voltage-gated potassium channel, and CYP2J2, a cytochrome P450. In contrast, margatoxin, a selective inhibitor of Kv1.3, which is expressed by macrophages, was without affect. Finally, RPMΦ from PECAM-1 –/– mice made less IL-6 in response to BTG, which was unaffected by DOF when compared with wild-type mice (Fig. 4a). These results correlate with those obtained for PMN recruitment (Table 1) and suggest that the ability of tissue-resident macrophages to secrete IL-6 was associated with PMN recruitment.

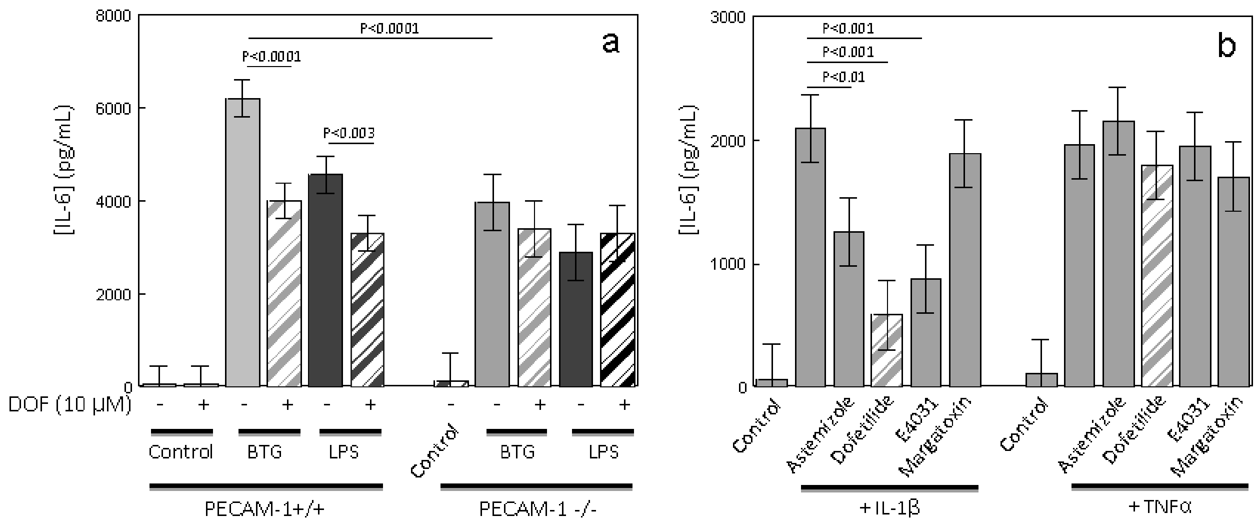

**FIGURE 4.** The effect of Kv11.1 inhibitors on IL-6 secretion by wild-type and PECAM-1 –/– RPMΦ. (a) Effect of DOF on IL-6 secretion by wild-type and PECAM-1 –/– RPMΦ in response to a variety of phlogistic agents. Supernatants were collected from PECAM-1 +/+ (n = 5) or PECAM-1 –/– (n = 3) RPMΦ cultured *in vitro* and exposed for 4 h to either BTG (1%) or LPS (10 ng/mL), where DOF (10 μM) may also have been present. Control represents results in the absence of an agonist. (b) Alternatively, RPMΦ from wild-type mice (n = 4) were cultured *in vitro* and exposed to either IL-1β (10 ng/mL) or TNFα (40 ng/mL) in the presence of either DOF (10 μM), E4031 (40 μM), astemizole (1 μM), or margatoxin (0. 1 μM). Levels of IL-6 were determined by ELISA (DuoSet). Data represent the mean ± 95% CI where no less than three replicates were performed for each individual mouse and where the supernatant was assayed neat and at 1:2 and 1:4 dilutions with final concentrations determined from the slope of a curve.

### Kv11.1 Expression by RPMΦ

Despite our best efforts, and in contrast to results obtained with human monocyte-derived macrophages, we have not been able to demonstrate conclusively the presence of Kv11.1 protein in RPMΦ by Western blot. Also of concern are our qPCR results, where it typically took 38–40 cycles before we saw message, while18S RNA was typically observed within 15–20 cycles. Separately, and in collaboration with M.J. Shipston (University of Edinburgh), and as also observed with human monocyte-derived macrophages, we failed to include or exclude a role for Kv11.1 by patch-clamp techniques[12]. Thus, we remain cautious before ascribing the pharmacological effects described to the specific inhibition of Kv11.1, although confirming the presence of Kv11.1 in RPMΦ remains a priority.

### IL-6 Expression by Wild-Type and PECAM-1 Knockout Macrophages Correlates with PMN Recruitment

To test the role of IL-6 in promulgating the inflammatory response and promoting leukocyte recruitment, we treated IL-6 –/– mice with BTG in a sterile model of peritonitis and observed that the number of extravasated PMNs was reduced compared to wild-type mice (Fig. 5a). Importantly, DOF had no additional effect on leukocyte recruitment in IL-6 –/– mice. Soluble gp130 inhibits murine IL-6 signaling[31]. Thus, to further test a role for IL-6 in PMN recruitment, we coadministered a soluble gp130 protein with DOF into the peritonea of both wild-type and PECAM-1 –/– mice prior to i.p. injection of BTG. In both instances, soluble gp130 was found to attenuate the inflammatory response to an equivalent degree (Fig. 5b). These results establish an important role for IL-6 in promoting leukocyte recruitment and suggest that the anti-inflammatory effect of DOF was causally linked to a suppression of inducible IL-6 expression.

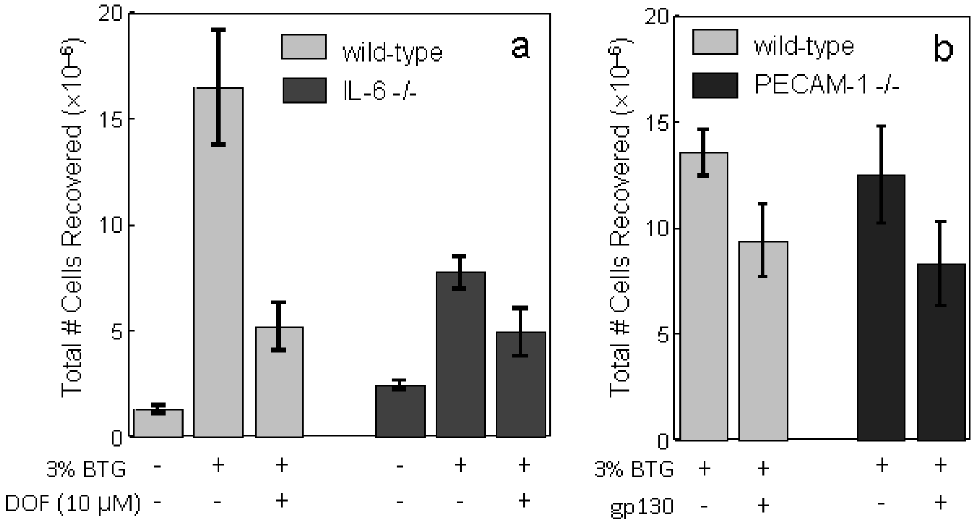

**FIGURE 5.** The importance of IL-6 in promoting leukocyte recruitment to the inflamed peritoneum. (a,b) Inflammatory cells were removed from the peritoneal cavity of wild-type, IL-6 –/–, or PECAM-1 –/– mice by lavage at 4 h after i.p. injection of BTG. Mice may also have received a single dose of DOF (10 mg/kg) (a) or a single dose of a soluble gp130 (250 ng/mouse) (b). Cell numbers were enumerated by hemocytometer for a minimum of six mice, each performed in triplicate. Data represent the mean ± 95% CI.

### C/EBPβ is Required for Dofetilide-Sensitive, LPS-Induced Expression of IL-6

LPS-induced IL-6 expression by RPMΦ from C/EBPβ –/– mice is impaired and C/EBPβ is reported to augment LPS-induced IL-6 and MCP-1 transcription[32,33]. Furthermore, robust transactivation of the proximal IL-6 promoter by C/EBPβ is known to be dependent on serine-64 of C/EBPβ[26]. To explore a role for serine-64, we used P388 murine B lymphoblasts, which lack endogenous C/EBPβ expression and are unresponsive to LPS for the expression of both IL-6 and MCP-1[33]. Transient or stable transfection of P388 cells with wild-type C/EBPβ can confer LPS-induced transcription of IL-6 and MCP-1, while a C/EBPβS64A mutant, which substitutes serine-64 with alanine (S64A), is greatly reduced in its capacity to support IL-6 and MCP-1 transcription[25,26]. Using P388 lymphoblasts stably transfected with either wild-type C/EBPβ or the C/EBPβS64A mutant, we isolated mRNA from cells that had been exposed to LPS ± DOF. IL-6 transcripts were measured in a radiolabeled RNase protection assay, revealing that DOF inhibited the ability of C/EBPβ to confer LPS-induced IL-6 expression, while having no effect on cells that expressed the C/EBPβS64A mutant (Fig. 6a–c). In addition, qPCR confirmed that P388 cells expressed message for Kv11.1 (data not presented).

These studies suggest that DOF regulates serine-64 phosphorylation of C/EBPβ. In order to test directly the ability of DOF to inhibit serine-64 phosphorylation, P388 cells that had been transiently transfected for C/EBPβ expression and stimulated by LPS were treated with and without DOF. Western blot analyses revealed that, while LPS stimulated the phosphorylation of serine-64 3.6-fold by 8 h poststimulation, the addition of DOF not only blocked LPS-induced phosphorylation, but also reduced serine-64 phosphorylation below basal levels (Fig. 6b).

### Dofetilide Inhibits Constitutive cdk2 Activity in Wild-Type, but not PECAM-1 –/– Mice

Phosphorylation of C/EBPβ at serine-64 is known to be mediated by cdk2[34]. To determine the effect of DOF on cdk2 activity, we cultured RPMΦ with 1% BTG, and collected cytosolic fractions from whole cell

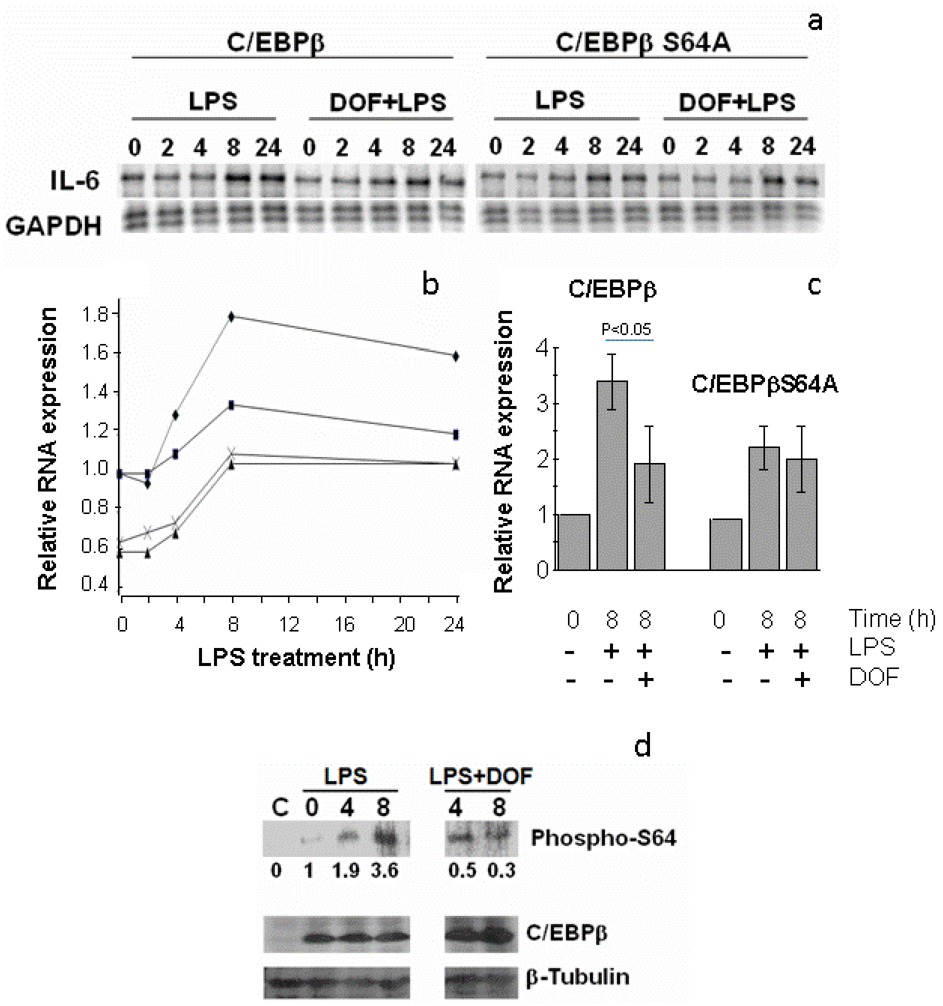

**FIGURE 6.** The effect of DOF on C/EBPβ-dependent IL-6 expression by P388 cells in response to LPS. (a,b) RNA protection assays for IL-6 were performed with RNA isolated from representative populations of P388 cells ectopically expressing either wild-type C/EBPβ (◆,■) or C/EBPβS64A (▲, **X**). Cells were treated with LPS (◆,▲) or LPS + DOF (■,**X**). RNA was isolated over the time course shown with 20 µg of RNA analyzed. (b) Quantitation of the RNase-resistant probe was performed with a Storm PhosphorImager (Molecular Dynamics, Sunnyvale, CA). The abundance of IL-6 mRNA was normalized to that of GAPDH in the same samples and set to a value of 1.0 for cells expressing wild-type C/EBPβ. Data represent the average of two separate experiments for which a representative RNase protection assay is shown. (c) The above experiments were repeated a further three times with IL-6 message analyzed by RPA, 8 h after LPS ± DOF treatment. (d) Western blot analyses were performed with total lysates isolated from representative populations of P388 cells transiently transfected for C/EBPβ expression. Cells were treated with LPS or LPS + DOF. The abundance of C/EBPβ serine-64 phosphorylation product was normalized to the abundance of total C/EBPβ, and set to a value of 1.0 for cells transfected for C/EBPβ expression and untreated with LPS. Detection of γ-tubulin is included as a loading control.

lysates over a 2-h time course. These samples were then analyzed and quantitated by Western blot using an antibody that recognized the activated phosphorylated form of cdk2. These studies demonstrate that DOF inhibited the constitutive activation of cdk2 in unstimulated cells, but failed to inhibit maximal induction of cdk2 activity with BTG as seen by 45 min (Fig. 7a,b). Similar results were obtained with LPS, IL-1β, and TNFα in which cdk2 activity was only monitored at the 45-min time point (Fig. 7c). Of particular interest, DOF had no inhibitory effect on the constitutive activation of cdk2 in resting PECAM-1 –/– RPMΦ (Fig. 7d).

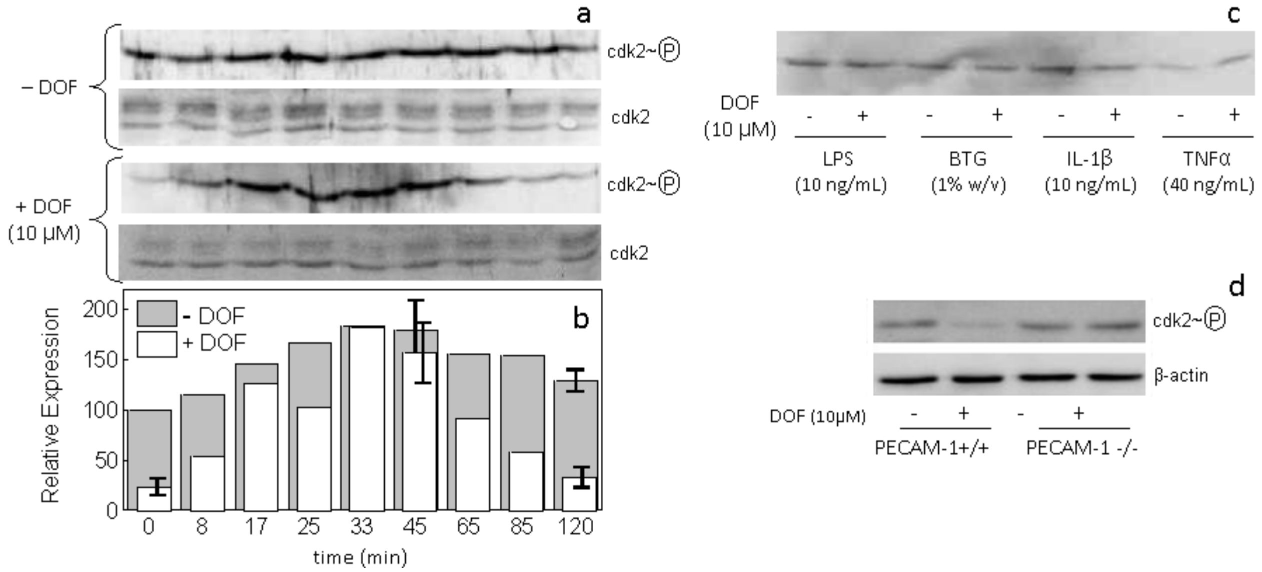

**FIGURE 7.** The effect of DOF on constitutive and agonist-induced phosphorylation of cdk2. (a) Western blot analysis for active cdk2 was performed on cytosolic fractions from whole cell lysates of RPMΦ following treatment with 1% BTG over the time course shown. In all cases, protein loading was based on equal numbers of cells extracted and where visual inspection of the transblots following Ponceau Red staining confirmed equivalent protein loading. Separate gels were run for either phospho-CDK2(Thr160) or total cdk2 in which transblots were also probed for β-actin. (b) Chemiluminescence in (a) was quantitated and normalized to the relative expression of untreated cells at t = 0 min. The experiment was repeated a further two times, but where cells were extracted only at 0, 45, and 120 min time points. Error bars represent 95% CI. (c) Western blot analysis for active cdk2 in cytosolic fractions prepared from RPMΦ treated with various agonists for 45 min either in the presence or absence of DOF (10 μM). (d) Western blot analysis for active cdk2 in cytosolic fractions from PECAM-1 +/+ or PECAM-1 –/– RPMΦ treated with or without DOF in the absence of any other stimulus. A β-actin blot is also shown as a control for loading and equal numbers of cells extracted.

### DISCUSSION

The importance of membrane electrophysiology in influencing the phlogistic responses of macrophages is well described but, from a mechanistic point of view, poorly defined[35,36,37,38,39]. Although many studies have used nonselective potassium channel inhibitors or have deliberately depolarized macrophages by exposing cells to high concentrations of extracellular $K^+$, very few studies have focused on the role of specific ion channel conductances. That said, pharmacological and selective inhibition of the large-conductance calcium- and voltage-activated potassium channel MaxiK (BK channel) with paxilline is known to inhibit both TLR/IL-1R, but not TNF-R ligand-induced activation of NF-κB, resulting in a suppression of both TNFα and IL-6 expression[40,41]. Precisely how MaxiK regulates NF-κB activation is not known, but patch-clamp analysis of excised macrophage membrane and single-channel conductance measurements support the view that potassium efflux through MaxiK in response to LPS is an early event in regulating the inflammatory response of human monocyte-derived macrophages.

The novel data, presented herein, reveal that selective inhibitors of Kv11.1, another voltage-gated potassium channel, can inhibit proinflammatory responses both *in vivo* and *in vitro*, having a significant effect on the ability of RPMΦ to express both IL-6 and MCP-1 inducibly, but not TNFα or the CXC chemokines KC and MIP-2. This contrasts with the effect of paxilline on hMDMΦ, which inhibits both IL-6 and TNFα without affecting the CXC chemokine IL-8. Although this subtle difference may be species specific, it may also suggest that the transcriptional activity of macrophages in response to proinflammatory stimuli can be fine tuned by individual voltage-gated potassium channels that may further be dependent on the activation state or phenotype of the macrophage being examined. The significance of these results is, as yet, not fully understood, but warrants further investigation. Nevertheless, it is recognized that macrophage phenotypes can be defined as much by the nature of their rectifying potassium currents as by the cytokines they express[12].

Evidence that a reduction in IL-6 may have been responsible for the absence of an inflammatory leukocyte infiltrate in sterile models of peritonitis was afforded using IL-6 –/– mice or wild-type mice treated with an IL-6 antagonist. Thus, an early reduction in TLR/IL-1R–induced IL-6 (and MCP-1) expression appeared to correlate strongly with a reduction in leukocyte recruitment. This is a conclusion wholly supported by observations made in other models of acute inflammation, including subcutaneous air pouch, carrageenan-induced pleurisy, collagen-induced arthritis, and SES-induced peritonitis[42,43,44]. Nevertheless, our conclusions are tempered by the finding that while DOF blocked PMN recruitment in peritonitis, induced IL-6 levels were typically reduced no more than 25% in wild-type mice. Considering the inability of DOF to inhibit induced IL-6 or MCP-1 expression by mesothelial cells, the results suggest that DOF may also affect other factors that regulate the inflammatory process. Thus, a reduction in IL-6 expression by activated macrophages may be a derivative of other, more subtle, transcriptional changes that we have not yet identified. These results, nevertheless, raised the question: How might a voltage-gated potassium channel regulate IL-6 expression?

Characterization of the human IL-6 promoter reveals binding elements for AP-1, C/EBPβ, CRE, and NF-κB transcription factors[45], but only in the case of the NF-κB binding element is there evidence that the expression of IL-6 can be uncoupled from TNFα in response to TLR/IL-1R ligands[46,47,48]. Whereas TNFα is driven predominantly via a classic p50-RelA NF-κB heterodimer complex, IL-6 can be driven by NF-κB p50 associating with an inducible IκBζ. It is therefore significant that IκBζ-deficient macrophages are known to manifest a severe impairment of IL-6 production, but not TNFα, in response to a variety of TLR ligands and IL-1[46]. Of particular relevance is the observation that phosphorylation of serine-64 of C/EBPβ is critical for potentiating the synergistic activity of C/EBPβ and NF-κB that drives IL-6 transcription[26]. Using a murine B lymphoblastic cell line, which does not normally express C/EBPβ, we were able to show, using stable transfectants, that DOF inhibited the ability of wild-type C/EBPβ, but not a serine-64 mutant, to induce IL-6 expression in response to LPS. Furthermore, DOF treatment was able to block LPS-induced phosphorylation of serine-64 completely. These results suggest that DOF can regulate IL-6 expression via serine-64 phosphorylation of C/EBPβ, although they do not exclude the possibility of other pathways being affected which may, or may not, impact on IL-6 expression.

Several kinases have been implicated in the phosphorylation of C/EBPβ, but only cdk2 has been shown to phosphorylate serine-64[34]. In this instance, cdk2 functioned as a downstream effector of Ras/Raf signaling, a pathway also activated in macrophages following exposure to LPS[49]. We therefore monitored the temporal activation of cdk2 following exposure of macrophages to LPS and observed that whereas DOF did not affect maximal cdk2 activity seen by 45 min, DOF did inhibit constitutive activation of cdk2. We consider this observation important for several reasons. First, inhibition of cdk2 with roscovitine is anti-inflammatory in several murine models of acute and chronic inflammation[50]. Although the authors of this study ascribed the *in vivo* effects of roscovitine to promoting cell death of extravasated PMNs, their data can also be explained by the suppression of IL-6, as these models are known to be IL-6 and C/EBPβ dependent[43,44,51]. Second, C/EBPβ is fully active in resting macrophages and poised to stimulate transcription in conjunction with other factors whose activities are transcriptionally induced[52], e.g., IκBζ[46]. Finally, membrane potential and membrane depolarization are reported to regulate cdk2 activity[53]. Precisely how membrane potential regulates cdk2 activity remains to be determined and is the focus of continued study.

We have previously demonstrated that antibody-mediated cross-linking or homophilic ligation of PECAM-1 can inhibit a DOF-sensitive repolarizing current in depolarized macrophages to promote β1 integrin adhesive events[6]. It is our contention that PECAM-1, by inhibiting Kv11.1, can function to inhibit macrophage expression of IL-6 in response to TLR/IL-1R ligands, perhaps in a β1 integrin–dependent manner. Indeed, IL-6 expression by thioglycollate-elicited peritoneal macrophages is modulated by β1 integrins and fibronectin[54], and Kv11.1 is part of a larger macromolecular signaling complex incorporating β1 integrins[8,9]. In the absence of PECAM-1 homophilic interactions, we might therefore expect that IL-6 levels would be raised in response to proinflammatory stimuli. This is indeed what is observed for PECAM-1 –/– mice in response to endotoxic shock, where plasma levels of IL-6

(and MCP-1) relative to wild-type mice are elevated, while TNFα induction remains comparable. It may also be relevant that inflammation and lipid accumulation in nonalcoholic hepatic steatosis, which is accentuated in the PECAM-1 –/– mouse, is also IL-6 and C/EBPβ dependent[24,55,56]. Finally, PECAM-1 –/– mice are characterized by increased endothelial barrier dysfunction in several models of inflammation, where under hypoxic conditions, we would expect augmented IL-6 expression to result in increased vascular permeability[57].

Our finding that DOF had only a marginal effect on IL-6 levels within the inflamed peritoneum is also consistent with mesothelial cells being a major source of IL-6 in the peritoneal tissue[58]. This might explain why PECAM-1 –/– mice on a C57BL/6 background do not manifest any obvious leukocyte recruitment defect. This conclusion, however, is tempered by our finding that resident peritoneal macrophages from PECAM-1 –/– mice were refractory to DOF with respect to IL-6 expression and constitutive cdk2 activity, suggesting that these cells had compensated for the loss of PECAM-1.

In conclusion, our data provide a novel mechanistic insight into the anti-inflammatory effects of class III antiarrhythmic methanesulfonanilides. In addition, our data also provide a possible mechanistic explanation for the discordant response seen in PECAM-1 –/– mice to IL-1β and TNFα in the cremaster model, while failing to show a difference in peritonitis.

### ACKNOWLEDGMENTS

This work was supported by the Wellcome Trust (064487 to S.B.B.), LRF (07044 to S.B.B.), and the American Heart Association, Midwest Affiliate (0755809Z to R.C.S.).

We are indebted to Michael (Spike) Clay and Aili Zhang for technical assistance. We are also indebted to Nicholas Topley (Cardiff) for helpful discussions and supply of IL-6 –/– mice. S.B.B., C.H., T.B.K., J.K, and D.C. did the experiments, while S.B.B. wrote the paper with the help of M.P, R.C.S.
